# High baseline Tie1 level predicts poor survival in metastatic breast cancer

**DOI:** 10.1186/s12885-019-5959-8

**Published:** 2019-07-24

**Authors:** Leena Tiainen, Emilia A. Korhonen, Veli-Matti Leppänen, Tiina Luukkaala, Mari Hämäläinen, Minna Tanner, Outi Lahdenperä, Pia Vihinen, Arja Jukkola, Peeter Karihtala, Sonja Aho, Eeva Moilanen, Kari Alitalo, Pirkko-Liisa Kellokumpu-Lehtinen

**Affiliations:** 1 Department of Oncology, Faculty of Medicine and Health Technology, Tampere University and Tays Cancer Centre, P.O. Box 100, FI-33014 Tampere, Finland; 2 Department of Oncology, Tampere University Hospital, P.O. Box 2000, FI-33521 Tampere, Finland; 3 Wihuri Research Institute and Translational Cancer Biology Program, University of Helsinki, Biomedicum Helsinki, P.O. Box 63, FI-00014 Helsinki, Finland; 4 Research, Development and Innovation Centre, Tampere University Hospital and Faculty of Medicine and Health Technology, Tampere University, P.O. Box 2000, FI-33521 Tampere, Finland; 5 The Immunopharmacology Research Group, Faculty of Medicine and Health Technology, Tampere University and Tampere University Hospital, P.O. Box 100, FI-33014 Tampere, Finland; 6 Department of Oncology and Radiotherapy, Turku University Central Hospital, P.O. Box 52, 20521 Turku, Finland; 7 Department of Oncology and Radiotherapy, Medical Research Center Oulu, Oulu University Hospital and University of Oulu, P.O. Box 10, 90029 OYS Oulu, Finland

**Keywords:** Tie1, Angiopoietin-2, Angiogenesis, Metastatic breast cancer, Prognostic marker

## Abstract

**Background:**

Angiopoietin growth factors (Angs) regulate angiogenesis and lymphangiogenesis by binding to the endothelial Tie2 receptor. Ang2 expression is elevated in tissue hypoxia and inflammation, which also induce cleavage of the extracellular domain of the orphan Tie1 receptor. Here we have examined if the concentrations of Ang2 and the soluble extracellular domain of Tie1 in patient plasma are associated with the prognosis of patients with metastatic breast cancer.

**Methods:**

Plasma Tie1 and Ang2 levels were measured in metastatic breast cancer patients treated in a phase II trial with a taxane-bevacizumab combination chemotherapy in the first-line treatment setting. They were analyzed before treatment, after 6 weeks and 6 months of treatment, and at the final study visit. Using the median concentrations as cutoffs, Tie1 and Ang2 data were dichotomized into low and high concentration groups. Additionally, we analyzed Tie1 concentrations in plasma from 10 healthy women participating in a breast cancer primary prevention study.

**Results:**

Plasma samples were available from 58 (89%) of the 65 patients treated in the trial. The baseline Tie1 levels of the healthy controls were significantly lower than those of the metastatic patients (*p* < 0.001). The overall survival of the patients with a high baseline Tie1 level was significantly shorter (multivariate HR 3.07, 95% CI 1.39–6.79, *p* = 0.005). Additionally, the progression-free survival was shorter for patients with a high baseline Tie1 level (multivariate HR 3.78, 95% CI 1.57–9.09, *p* = 0.003). In contrast, the baseline Ang2 levels had no prognostic impact in a multivariate Cox proportional hazard regression analysis. The combined analysis of baseline Tie1 and Ang2 levels revealed that patients with both high Tie1 and high Ang2 baseline levels had a significantly shorter overall survival than the patients with low baseline levels of both markers (multivariate HR for overall survival 4.32, 95% CI 1.44–12.94, *p* = 0.009).

**Conclusions:**

This is the first study to demonstrate the prognostic value of baseline Tie1 plasma concentration in patients with metastatic breast cancer. Combined with the results of the Ang2 analyses, the patients with both high Tie1 and Ang2 levels before treatment had the poorest survival.

**Trial registration:**

Clinicaltrials.gov: NCT00979641, registration date 19-DEC-2008. The regional Ethics Committee: R08142M, registration date 18-NOV-2008.

## Background

Several drugs targeting the vascular endothelial growth factor (VEGF) and VEGF receptors (VEGFR) are currently used as treatment of various cancer types in clinics [1]. VEGF targeting antibodies alleviate age-related macular degeneration, but in cancer therapy, they provide only limited benefits. For this reason, a significant interest has emerged in the targeting of the more recently characterized Ang/Tie endothelial growth factor system, which has essential functions in embryonic development, the regeneration of the mature vasculature, tissue inflammation and tumor angiogenesis [2–6]. Angiopoietin growth factors (Ang1–4) bind to the Tie2 receptor. The homologous Tie1 protein does not bind angiopoietins directly, although it participates in the Ang-Tie2 signal transduction complex [2, 6–8].

Ang1 stabilizes the vasculature after angiogenesis and is a more potent Tie2 agonist than Ang2, which can act as an agonist or antagonist of the Tie2 receptor, depending on a number of other factors [9–11]. In normal homeostasis, Ang2 levels are low, but the Ang2/Ang1 ratio is increased in inflamed tissues, e.g. in sepsis and in malignancies, including breast cancer [12, 13]. High Ang2 levels are associated with poor patient survival in multiple malignancies, breast cancer among others [14–19]. Some of the Ang/Tie system targeted antibodies have already been evaluated in clinical trials, but so far, the effects of anti-Ang2 monotherapy have been modest [20–22]. A better understanding of Ang function is clearly needed for the rational development of effective Ang-pathway targeted therapies. Although Tie1 expression in endothelial cells is increased in tumor vessels and deletion of the Tie1 gene in tumor-bearing mice decreased tumor growth and angiogenesis in preclinical experiments [4, 23], the significance of Tie1 in tumor progression is also unclear. Tie1 ectodomain cleavage occurs in vivo in association with acute [11] and chronic inflammation [24], leading to increased concentration of the soluble extracellular domain in the serum of patients with severe viral infections [11]. Furthermore, Tie1 deletion in a murine metastasis model tightened endothelial barrier and therefore, reduced metastatic foci [25].

In the present study, we investigated the prognostic value of the circulating levels of Tie1 and Ang2 in patients who received first-line taxane-bevacizumab combination -chemotherapy combination for the treatment of metastatic breast cancer. Additionally, we explored if a combined analysis of Tie1 and Ang2 levels would help to identify the patients with poor prognosis in need of novel treatment approaches.

## Methods

All together 65 patients with histologically verified HER2-negative advanced breast cancer were enrolled into the single-arm, prospective, phase 2 study in three Finnish university cancer clinics between May 2009 and October 2013 (NCT00979641). The method of patient recruitment, the study design and the clinical trial results were previously published [26]. Briefly, patients included in the study received a taxane (paclitaxel 90 mg/m^2^ on days 1, 8 and 15 or docetaxel 50 mg/m^2^ on days 1 and 15) with bevacizumab (10 mg/kg on days 1 and 15) on a treatment cycle of 4 weeks as the first-line chemotherapy for metastatic breast cancer. Docetaxel was given to 32 patients and 33 patients received paclitaxel.

Bevacizumab 15 mg/kg every three weeks was continued as maintenance therapy for those patients with non-progressive disease after taxane discontinuation. In addition to bevacizumab, patients with hormone receptor-positive disease received endocrine therapy. Furthermore, bevacizumab could be continued with second-line chemotherapy. All patients provided written informed consent and the regional Ethics Committee approved the study protocol (R08142M).

Blood samples were obtained from the patients during treatment. EDTA samples for plasma analysis were obtained at the baseline, every 6 weeks during the bevacizumab-taxane combination, at the discontinuation of taxane treatment, during the bevacizumab maintenance therapy, first every three weeks for the first two months and thereafter every 12 weeks, and at the final study visit.

Healthy control samples were obtained from 10 women participating in a mammography screening program at the Hatanpää Breast Clinic in Tampere. These women voluntarily participated in a breast cancer primary prevention study currently in progress at the University of Tampere and, as a part of the accepted protocol, blood samples were drawn for scientific purposes. All participants gave their written informed consent and the regional Ethics Committee approved the study (R15023).

### Measurement of plasma Tie1 and Ang2 levels by ELISA assay

Tie1 and Ang2 levels were measured in patient plasma samples using a modified hTie1 and hAng2 ELISA protocol (R&D Systems Europe Ltd., Abingdon, UK, Duoset, DY5907 and DY623, respectively). Briefly, a 96-well plate was coated with 100 μl of diluted capture antibody (1:180 in PBS) per well and incubated o/n at room temperature (RT). The wells were washed three times with PBS-0.05% Tween 20, followed by blocking with 300 μl/well of the Reagent Diluent 2 (R&D, Y995) for 1.5 h at RT on an orbital shaker for Tie1 or with 250 μl/well of 1% BSA in PBS for 1 h at RT for Ang2. The wells were washed 3 x with PBS-0.05% Tween 20. For Tie1, 50 μl/well of the reagent RD1–89 (R&D, DILUENT08) was added. Standards and samples diluted in the RD5–17 reagent (R&D, RD508) were pipetted into the wells at 100 μl/well and incubated for 2 h at RT on an orbital shaker. For Ang2, 100 μl of a sample or standards in diluent reagent (1% BSA in PBS) was added to the wells, and incubated for 2 h at RT. The wells were washed 3 x with PBS-0.05% Tween 20 and 100 μl of detection antibody diluted 1:180 in diluent reagent (Reagent Diluent 2, R&D, for Tie1, 1% BSA in PBS for Ang2) was added and incubated for 2 h at RT on an orbital shaker for Tie1 and for 1.5 h at RT for Ang2. After washing 3 x with PBS-0.05% Tween 20, 100 ul of SA-HRP solution per well (in Reagent Diluent 2 for Tie1, and in 1% BSA in PBS for Ang2) was added before incubating for 20 min at RT. The wells were washed 3 x with PBS-0.05% Tween 20. Then a mixture of Color Reagent A and Color Reagent B for Tie1 (R&D, DY999) and BioFX®TMB substrate solution for Ang2 (SurModics, Eden Prairie, MN, USA) was added at 100 μl/well and incubated for 20 min in the dark at RT. Stop solution (50 μl of 1 M HCl) was added, and the absorbance of each well was measured within 20–30 min using a microplate reader with the filter set to 450 nm and the correction wavelength set to 540 nm. The interassay coefficients of variation for Tie1 and Ang2 were 11.4 and 7.1%, respectively.

### Patient characteristics

**Fig. 1:**
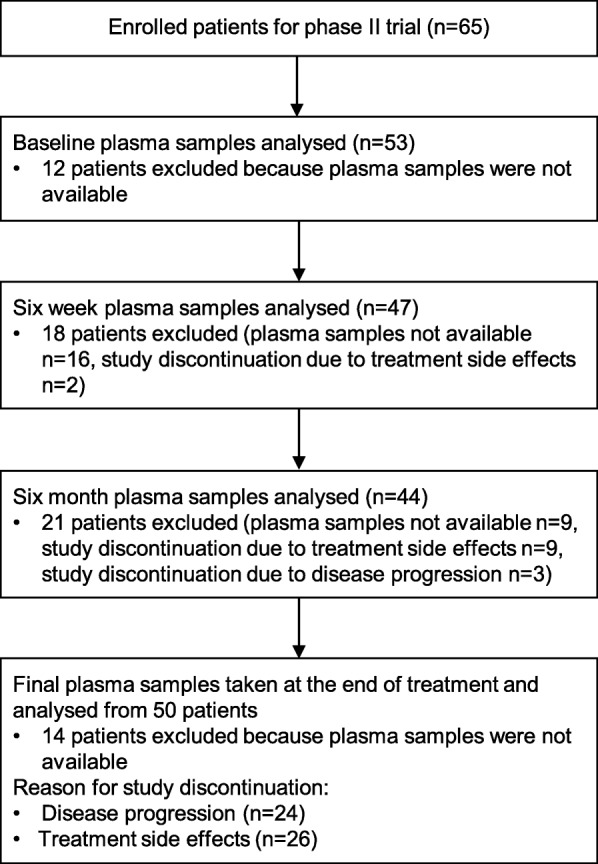
Flow diagram of study patient plasma samples analyzed at baseline, after six weeks’ treatment, after six months’ treatment and at final visit. Final plasma samples were taken at the final study visit and the reasons for discontinuation are presented in the chart

The patient population and the analyzed plasma samples were identical to our previous paper focusing on plasma interleukin-8 levels as a prognostic marker [27]. At the baseline, plasma samples were available from 53 patients (82%). Overall, plasma samples were available from 58 (89%) of the 65 patients treated in the study. Key characteristics of the study population and the main efficacy outcomes are presented in Table 1. Plasma samples for Tie1 and Ang2 were analyzed at four time points: at the baseline, six weeks after the treatment initiation, six months after the treatment initiation and at the final visit. The number of patients that had plasma samples analyzed and the reasons for exclusions are presented in a flow chart (Fig. 1). Six weeks’ and six months’ samples were available only for those patients that were still on study treatment at that time point.

**Table 1:** Baseline characteristics and the efficacy outcomes of the plasma biomarker population compared to the overall study population

	Plasma biomarker population (*n* = 58)	Overall study population (*n* = 65)
Age, years		
Median (range)	58 (32–75)	57 (32–75)
Menopausal status, n (%)		
Pre-menopausal	9 (15.5)	10 (15.4)
Post-menopausal	49 (84.5)	55 (84.6)
History of early stage disease, n (%)	52 (89.7)	57 (87.7)
Disease free interval, n (%)		
≤ 24 months	10 (19.2)	11 (19.3)
> 24 months	42 (80.8)	46 (80.7)
Hormone receptor status, n (%)		
ER+ and/or PR+	47 (81.0)	53 (81.5)
ER- and PR-	11 (19.0)	12 (18.5)
Number of metastatic lesions, n (%)		
≤ 3	11 (19.0)	14 (21.5)
> 3	47 (81.0)	51 (78.5)
Extent of disease		
< 3 sites	36 (62.1)	39 (60.0)
≥ 3 sites	22 (37.9)	26 (40.0)
Site of metastatic disease, n (%)		
Visceral disease	46 (79.3)	53 (81.5)
Non-visceral disease	12 (20.7)	12 (18.5)
Median overall survival, months (95% CI)	37.5 (25.4–49.6)	35.1 (22.2–50.3)
Median progression-free survival, months (95% CI)	11.3 (8.3–14.4)	11.3 (9.7–16.0)
Response to treatment		
Complete response/partial response	38 (71.7)	40 (61.5)
Stable disease	13 (24.5)	15 (23.1)
Progressive disease	2 (3.8)	3 (4.6)

The median progression-free survival (PFS) and overall survival (OS) were similar for patients treated with docetaxel or paclitaxel (PFS: *p* = 0.47, OS: *p* = 0.77). The median OS for patients with triple-negative breast cancer was 17.9 months (95% CI 8.5–26.9). Furthermore, the median OS for patients with hormone receptor positive metastatic breast cancer was 45.0 months (95% CI 30.2–51.3).

The mean age of the ten healthy controls was 57.8 years (range 54–67).

### Statistical analysis

The statistical plan for the biomarker analysis was exploratory. Tie1 and Ang2 were dichotomized as low or high for each patient using the median value as the cutoff. Sensitivity, specificity and area under curve (AUC) for plasma Tie1 concentration were determined using receiver operator characteristic (ROC) analysis. Baseline Tie1 or Ang2 levels as independent prognostic factors (below/above median) were evaluated using Cox proportional hazard regression analysis. Multivariate analysis was performed using the Cox model, and it was adjusted by age (continuous), menopausal status (yes/no), hormone receptor status (negative/positive), presence of visceral metastasis (yes/no), number of metastatic lesions (cut-off of three metastatic lesions) and extent of the disease (cut-off of three metastatic sites). The Mann-Whitney U test was used to compare differences in the baseline Tie1 and Ang2 levels between groups with different baseline characteristics. The Wilcoxon signed rank test was used to compare between baseline and week 6 plasma Tie1 and Ang2 levels. *P*-values under 0.05 were considered statistically significant. Statistical analyses were performed using SPSS version 23 statistical software package (SPSS Inc., Chicago, IL, USA).

## Results

### Tie1 and Ang2 plasma levels

**Fig. 2:**
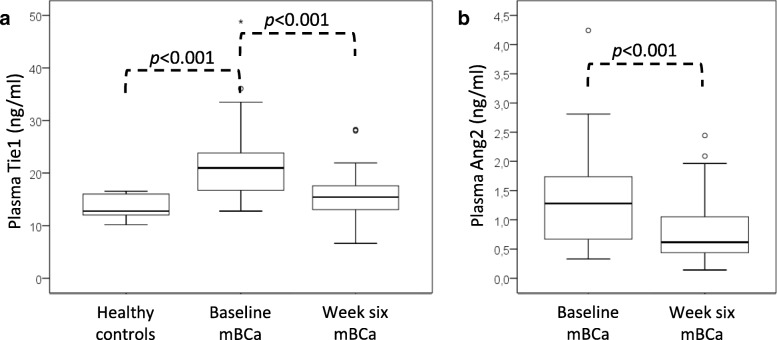
Plasma Tie1 and Ang2 levels. **a** Tie1 levels of healthy controls compared to the Tie1 levels of patients with metastatic breast cancer at baseline and at week six after treatment. **b** Ang2 levels of patients with metastatic breast cancer at baseline and at week six after treatment. mBCa = metastatic breast cancer

For the study population, the median Tie1 level at baseline was 21.0 ng/ml (95% CI 17.8–23.3, Fig. 2a), and the median Ang2 level at baseline was 1.29 ng/ml (95% CI 1.03–1.52, Fig. 2b). The baseline Tie1 levels were significantly lower in the healthy controls than in the metastatic breast cancer patients (Fig. 2a). The median Tie1 level for healthy controls was 12.8 ng/ml (95% CI 10.4–16.5, Fig. 2a). The most optimal cut-off value (16.0 ng/ml) for plasma Tie1 concentration had a sensitivity of 77.4%, but a specificity of only 30.0%, for distinguishing metastatic breast cancer patients from healthy controls with an AUC 0.917 (95% CI 0.839–0.995, *p* < 0.001).

There were no statistically significant differences in baseline Tie1 or Ang2 levels between groups with different baseline characteristics, including menopausal status (*p* = 0.09 for Tie1, *p* = 0.13 for Ang2), hormone receptor status (*p* = 0.80 for Tie1, *p* = 0.14 for Ang2), number of metastatic lesions (*p* = 0.69 for Tie1, *p* = 0.37 for Ang2) or visceral disease (*p* = 0.92 for Tie1, *p* = 0.15 for Ang2). Only the patients with more than three metastatic sites had significantly higher baseline Tie1 levels than the patients with fewer metastatic sites (median Tie1 23.7 ng/ml, 95% CI 21.0–29.0 vs. 17.8 ng/ml, 95% CI 16.0–21.1, *p* = 0.002). Similarly, the patients with more than three metastatic sites had significantly higher baseline Ang2 levels (median Ang2 1.08 ng/ml, 95% CI 0.66–1.36 vs. 1.54 ng/ml, 95% CI 1.23–2.29, *p* = 0.008).

Differences in Tie1 and Ang2 concentrations between baseline and week six samples were analyzed to evaluate the treatment effect. The median baseline Tie1 level was 21.0 ng/ml (95% CI 17.8–23.3), which was significantly higher than the median Tie1 level at six weeks (15.4 ng/ml [95% CI 14.1–17.1], *p* < 0.001, Fig. 2a). The median decrease in the Tie1 level between these two time points was 22.9% (95% CI 20.9–27.4). The median baseline Ang2 level was 1.29 ng/ml (95% CI 1.03–1.52) and the median Ang2 level at six weeks was 0.62 ng/ml (95% CI 0.57–0.84). The median decrease in the levels of Ang2 from the baseline to six weeks, 47.0% (95% CI 34.5–52.9), was also statistically significant (*p* < 0.001, Fig. 2b).

### Effect of Tie1 or Ang2 levels on survival

**Fig. 3:**
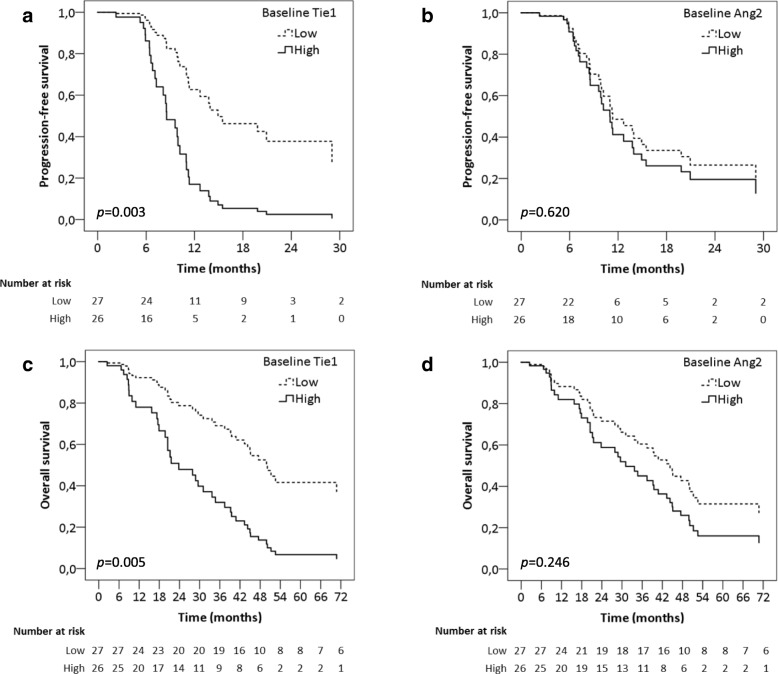
Progression-free survivals and overall survivals grouped by baseline plasma Tie1 or Ang2 levels **a** Progression-free survival for patients with high or low plasma Tie1 at baseline. **b** Progression-free survival for patients with high or low plasma Ang2 at baseline. **c** Overall survival for patients with high or low plasma Tie1 at baseline. **d** Overall survival for patients with high or low plasma Ang2 at baseline. Cox regression analysis adjusted by age, menopausal status, hormone receptor status, presence of visceral metastasis, number of metastatic lesions and extent of the disease

Median progression-free survival was longer for patients in the low baseline Tie1 level group than for the patients in the high baseline Tie1 group (Fig. 3a, Table 2). No difference was observed in progression-free survival in relation to baseline Ang2 levels (Fig. 3b, Table 2).

**Table 2:** Cox regression analysis for progression-free survival

	N	n	Progression-free survival			
			Age-adjusted HR [95% CI]	*p* value	multivariate HR^a^ [95% CI]	*p* value
Baseline Tie1						
Low	27	16	1		1	
High	26	15	**2.13 [1.02–4.46]**	**0.043**	**3.78 [1.57–9.09]**	**0.003**
Baseline Ang2						
Low	27	14	1		1	
High	26	17	1.21 [0.59–2.47]	0.597	1.22 [0.54–2.77]	0.620
Combined analysis						
Tie1 and Ang2 low	18	9	1		1	
Tie1 low, Ang2 high	9	7	1.27 [0.47–3.43]	0.632	1.16 [0.39–3.39]	0.783
Tie1 high, Ang2 low	9	5	**3.86 [1.18–12.57]**	**0.025**	**4.45 [1.25–15.79]**	**0.021**
Tie1 and Ang2 high	17	10	2.02 [0.80–5.07]	0.133	**3.88 [1.25–12.06]**	**0.019**

Abbreviations: *N* number of patients, *n* number of events, *HR* hazard ratio, *CI* confidence interval. Statistically significant results are highlighted in bold

^a^Hazard ratio adjusted by age, menopausal status, hormone receptor status, presence of visceral metastasis, number of metastatic lesions and extent of the disease

The overall survival was significantly shorter for patients with a high baseline Tie1 concentration (Fig. 3c, Table 3). Additionally, patients with high baseline Ang2 levels had shorter overall survival when analyzed by the age-adjusted Cox hazard regression model (Table 3). However, in a multivariate Cox model adjusted by age, menopausal status, hormone receptor status, presence of visceral metastases, number of metastatic lesions and extent of disease, a high baseline levels of Ang2 alone was not a significant factor for poor prognosis (Fig. 3d, Table 3).

**Table 3:** Cox regression analysis for overall survival

	N	n	Overall survival			
			Age-adjusted HR [95% CI]	*p* value	multivariate HR^a^ [95% CI]	*p* value
Baseline Tie1						
Low	27	15	1		1	
High	26	24	**2.82 [1.41–5.66]**	**0.003**	**3.07 [1.39–6.79]**	**0.005**
Baseline Ang2						
Low	27	15	1		1	
High	26	24	**2.33 [1.20–4.54]**	**0.012**	1.58 [0.72–3.46]	0.246
Combined analysis						
Tie1 and Ang2 low	18	8	1		1	
Tie1 low, Ang2 high	9	7	2.21 [0.78–6.25]	0.135	1.34 [0.42–4.22]	0.612
Tie1 high, Ang2 low	9	7	2.77 [0.95–8.09]	0.062	2.73 [0.88–8.46]	0.080
Tie1 and Ang2 high	17	17	**4.79 [1.93–11.90]**	**0.001**	**4.32 [1.44–12.94]**	**0.009**

Abbreviations: *N* number of patients, *n* number of events, *HR* hazard ratio, *CI* confidence interval. Statistically significant results are highlighted in bold

^a^Hazard ratio adjusted by age, menopausal status, hormone receptor status, presence of visceral metastasis, number of metastatic lesions and extent of the disease

### Effect of combined analysis of Tie1 and Ang2 levels on survival

**Fig. 4:**
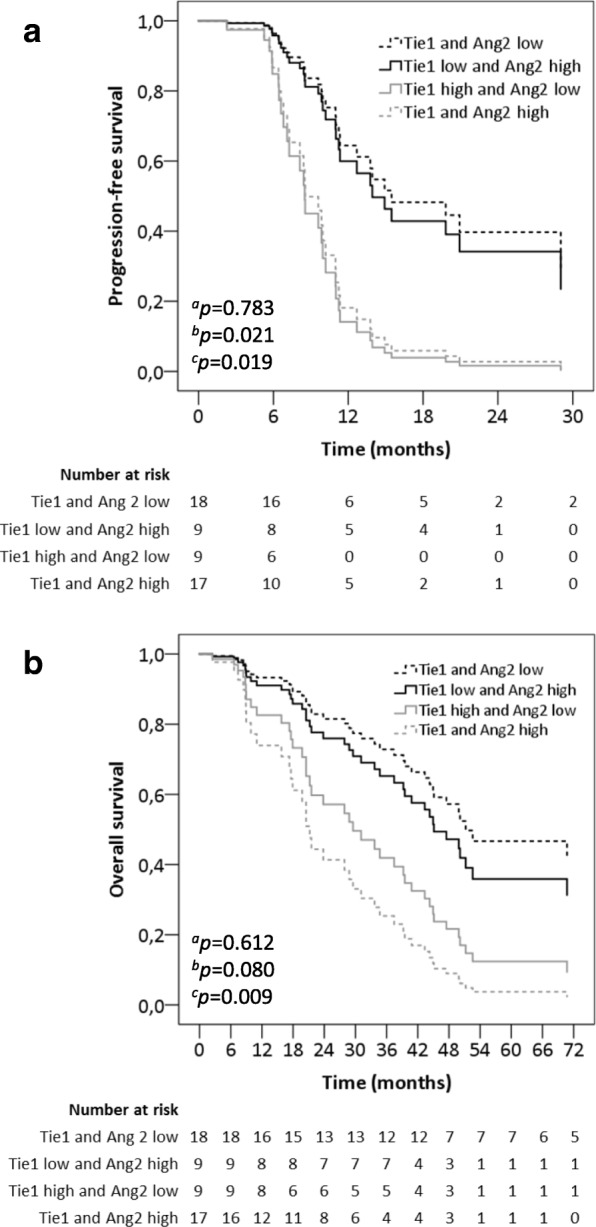
Progression-free survival and overall survival grouped by combined analysis of baseline plasma Tie1 and baseline plasma Ang2 levels. **a** Progression-free survival and **b** Overall survival for patients with high or low baseline Tie1 and Ang2. Cox regression analysis adjusted by age, menopausal status, hormone receptor status, presence of visceral metastasis, number of metastatic lesions and extent of the disease. ^a^*p*-value between Tie1 low, Ang2 low and Tie1 low, Ang2 high, ^b^*p*-value between Tie1 low, Ang2 low and Tie1 high, Ang2 low, ^c^*p*-value between Tie1 low, Ang2 low and Tie1 high, Ang2 high

For progression-free survival, the combined analysis of baseline Tie1 and Ang2 levels did not add any value compared to the Tie1 analysis on its own (Fig. 4a, Table 2). However, the combined analysis for high or low baseline Tie1 and Ang2 levels was more effective in the selection of patients with better overall survival (Fig. 4b, Table 3). The median overall survival for patients with low baseline levels of both Tie1 and Ang2 was 46.8 months (95% CI 23.8–79.8). In contrast, the median overall survival for patients with high baseline levels of both Tie1 and Ang2 was only 21.5 months (95% CI 8.8–34.7).

### Changes in plasma tie 1 or Ang2 levels and prognosis

The median decline in Ang2 levels between baseline and week six was 47.0%. The patients with Ang2 level decline higher than the median value had significantly worse prognoses. Multivariate Hazard Ratio (HR) for overall survival was 4.53 (95% CI 1.82–11.27, *p* = 0.001). In contrast, a high Tie1 decline during the first six weeks of treatment was not prognostic. The median Tie1 decline during this time period was 22.9%. The patients had similar survival whether they had Tie1 decline higher or lower than median value between baseline and week six (multivariate HR for overall survival 1.04, 95% CI 0.46–2.33, *p* = 0.921).

Only seven patients, i.e. 14% of the patients whose final samples were available, had at least 30% increased Tie1 plasma concentrations at their final visits, when compared to the previous measurements in each patient. For all these patients, the reason for study discontinuation was disease progression. Nevertheless, these patients had a similar overall survival as the patients with stable or declining Tie1 levels (multivariate HR 2.30, 95% CI 0.90–5.85, *p* = 0.078). At least 30% increased Ang2 concentration was observed in 24 patients at their final visits (48% of the patients whose final samples were available). The overall survival of these patients was significantly worse than in the patients with stable or declining Ang2 values (multivariate HR 2.17, 95% CI 1.09–4.31, *p* = 0.027).

## Discussion

The baseline concentration of the extracellular fragment of the orphan Tie1 receptor in bevacizumab plus taxane-treated breast carcinoma patients was found to be associated with both their overall survival and their progression-free survival (multivariate HR for overall survival 3.07, 95% CI 1.39–6.79, *p* = 0.005, multivariate HR for progression-free survival 3.78, 95% CI 1.57–9.09, *p* = 0.003). Previous studies have reported strong Tie1 expression in malignant tissues, including breast cancer [23, 28–30]. In gastric cancer, patients with Tie1 expression in their formalin-embedded tissue specimens had worse survival than the patients without Tie1 expression [30]. However, the prognostic value of circulating Tie1 levels has not been previously studied in malignant diseases.

Metastatic breast cancer patients had significantly higher baseline plasma Tie1 levels than the healthy controls (*p* < 0.001). However, circulating Ang2 levels are known to be higher on cancer patients [31] and therefore, we did not analyze plasma Ang2 levels on healthy controls.

Previous studies have indicated that the high concentration of the circulating Tie2 ligand Ang2 is associated with poor patient prognosis [16–19], and Ang2/Tie system-targeting antibodies and tyrosine kinase inhibitors are currently in clinical trials, including in those focused on breast cancer [20, 22]. In our study however, the baseline Ang2 level was not a significant prognostic marker for either progression-free survival or overall survival. However, it has been reported that an increase in serum Ang2 concentration during anti-VEGF treatment contributes to acquired drug resistance [32]. In our study, for the final plasma samples, a cut-off point of 30% was chosen because it was considered as a clinically meaningful change. In half of the patients of our study, the Ang2 plasma concentration was the highest at their final visit, and these patients had poor overall survival (multivariate HR 2.17, 95% CI 1.09–4.31, *p* = 0.027), perhaps because of increased acquired tumor chemoresistance [32].

Targeting of both Tie1 and Ang2 would be an interesting trial approach in the future for the treatment of breast cancer. In our study, high baseline Tie1 and Ang2 concentrations were associated with median overall survival of only 21.5 months (95% CI 8.8–34.7). This was significantly less than in the patients who had low plasma concentrations of both Tie1 and Ang2 (46.8 months, 95% CI 23.8–79.8, *p* = 0.009). Interestingly, additive inhibition of tumor growth was observed when angiopoietin activity was blocked in Tie1-deficient mice [4]. The possible synergistic effect of dual inhibition of Tie1 and Ang2 might be due to Ang2 influencing earlier phase in tumor growth than Tie1 [25].

According to our study, high baseline Tie1 level appears to be the best way to find the patients with short progression-free survival. In fact, the baseline Ang2 level and the combined analysis of Tie1 and Ang2 baseline levels do not provide additional information in terms of progression-free survival compared to Tie1 levels alone.

The Tie1 levels in healthy individuals were lower than in patients with metastatic disease before chemotherapy. During the bevacizumab and taxane therapy, the Tie1 levels declined substantially. However, the decline in Tie1 concentration was not related to the patient survival. Only the decrease in Ang2 concentration was prognostic, with a multivariate hazard ratio of 4.53 (95% CI 1.82–11.27, *p* = 0.001).

Bevacizumab has been investigated in several phase III trials as treatment of metastatic breast cancer. However, none of the trials has proven overall survival advantage for patients treated with bevacizumab [33]. Therefore, bevacizumab in only recommended for the treatment of highly selected patients with a need of a tumor response more commonly achieved with bevacizumab [33, 34]. All of our study patients were treated with bevacizumab. However, the effect of bevacizumab to Tie1 levels remains unexplored in this study. However, the main finding of our study was the prognostic value of pretreatment circulating Tie1 levels and bevacizumab did not confound this analysis.

Although, to our knowledge, this is the first study to evaluate the prognostic role of plasma Tie1 levels in breast cancer patients, the study has some limitations. Our study is a single-arm study with no control arm, and thus, the impact of bevacizumab on patient survival and the Tie1 and Ang2 concentrations during therapy cannot be evaluated. Furthermore, the study population size is limited, and therefore, our findings must be validated in a larger patient cohort.

Although immunohistochemical staining of Tie1 in tumor samples is associated with poor patient survival in breast cancer [35], the availability of tissue samples from metastatic tumors varies depending on tumor location, tumor load and the clinical need to accept the complication risks and discomfort related to needle aspirations. Circulating prognostic markers are more useful, and thus, high baseline circulating Tie1 and Ang2 levels before and during the treatment can be an additional way to identify patients with poor prognoses in this patient population, regardless of standard clinical characteristics. Most such patients do not derive a long-term benefit from the current chemotherapy treatment options. Novel treatment approaches, for example immunotherapies, are entering the clinics for many malignant diseases, and patients with poor prognoses should increasingly be referred to clinical trials. In preclinical studies, anti-angiogenic drugs and immune checkpoint inhibitors have demonstrated synergistic benefits [36], and they should be further studied in prospective clinical trials.

## Conclusions

High baseline plasma Tie1 level is a promising prognostic marker for both poor progression-free survival and for poor overall survival in metastatic breast patients treated with bevacizumab-taxane combination. The predictive value of circulating Tie1 levels should be evaluated in prospective clinical trials.

## Data Availability

The datasets used and analyzed during the current study are available from the corresponding author upon reasonable request.

## Abbreviations

Ang: Angiopoietin
Ang2: Angiopoietin-2
CI: Confidence interval
HR: Hazard ratio
RT: Room temperature
VEGF: Vascular endothelial growth factor
VEGFR: Vascular endothelial growth factor receptor
